# Development of EGFR TKIs and Options to Manage Resistance of Third-Generation EGFR TKI Osimertinib: Conventional Ways and Immune Checkpoint Inhibitors

**DOI:** 10.3389/fonc.2020.602762

**Published:** 2020-12-18

**Authors:** Leilei Wu, Linping Ke, Zhenshan Zhang, Jinming Yu, Xue Meng

**Affiliations:** ^1^ Department of Radiation Oncology, School of Medicine, Shandong University, Jinan, China; ^2^ Department of Radiation Oncology, Shandong Cancer Hospital and Institute, Shandong First Medical University and Shandong Academy of Medical Sciences, Jinan, China

**Keywords:** EGFR TKI resistance, osimertinib, combination (combined) therapy, immune check inhibitor, mechanisms of resistance, tumor immune environment, molecular biomarkers

## Abstract

Epidermal growth factor receptor tyrosine kinase inhibitors (EGFR TKIs) have been first-line therapy in the treatment of non-small cell lung cancer (NSCLC) harboring *EGFR* sensitive mutations. Progression inevitably happens after 10–14 months of first- or second-generation EGFR TKIs treatment for acquired resistance. Owing to the successful identification of *EGFR* T790M, third-generation EGFR TKIs such as osimertinib were developed to target such resistance mutation. Nowadays, osimertinib has shown its efficacy both in first-line and second-line after resistance to previous generations of TKI treatment of *EGFR*-mutant NSCLC. However, drug resistance also emerges on third-generation EGFR TKIs. Multiple mechanisms of acquired resistance have been identified, and some novel strategies were reported to overcome third-generation TKI resistance. Immune checkpoint inhibitors (ICIs) have dramatically changed the prognosis of selected patients. For patients with *EGFR*-addicted metastatic NSCLC, ICIs have also revealed a potential role. In this review, we will take stock of mechanisms of acquired resistance to third-generation TKIs and discuss current challenges and future perspectives in clinical practice.

## Introduction

Somatic alterations in epidermal growth factor receptor (EGFR) lead to abnormal activation of receptor tyrosine kinases (RTKs) signaling and occur in approximately 50% of Asian non-small cell lung cancer (NSCLC) patients and 15% (10–16.6%) of Caucasian NSCLC patients (1–6). EGFR, encoded by *EGFR*, also referred to as ErbB1/HER1, a member of human epidermal growth factor receptor (HER)/ErbB family, is a transmembrane RTK, followed by ErbB2 (HER2/neu), ErbB3 (HER3), and ErbB4 (HER4) (1, 7). Exons 18–24 encode the EGFR kinase domain and the most common alterations are deletions in exon 19 (ex19del, about 44%) and point mutations in exon 21 (L858R, about 41%), known as common mutations or classical mutations (1, 4, 8). Uncommon *EGFR* mutations in exons 18–21, such as L861X, G719X, and S768I, account for approximately 10% of *EGFR* mutations, also called atypical *EGFR* mutations (9, 10). These mutations increase activity of EGFR and then activate three main downstream signaling pathways: mitogen-activated protein kinases (MAPK)/extracellular signal-regulated kinases (ERK), phosphatidylinositol 3-kinase (PI3K)/AKT/mTOR, and interleukin 6 (IL-6)/Janus kinase (JAK)/signal transducer and activator of transcription 3 (STAT3) signaling pathways (7, 8, 11). These signaling cascades relate RTK activity to increased proliferation, motility, migration, survival, and anti-apoptotic cellular responses and facilitate genesis and development of NSCLC (8, 11).

In first-line treatment, first-generation EGFR TKIs erlotinib, gefitinib, and icotinib and second-generation afatinib and dacomitinib have exhibited advantages over various platinum-based chemotherapy in phase III trials for the treatment of patients with advanced NSCLC with activating *EGFR* mutations. However, most patients treated with first and second-generation EGFR TKIs inevitably develop acquired resistance through various mechanisms after a median period of 10–14 months (9, 12, 13). The most common mechanism is *EGFR* T790M mutation in exon 20 which accounts for approximately 50% of all EGFR TKIs resistance in NSCLC patients, more than half of which arises from T790M mutation in exon 20 (12, 13). In summary, the two generations are considered ineffective in management of T790M-positive NSCLC although second-generation shows some special effects. In recent years, multiple third-generation mutation-selective EGFR TKIs have been developed to overcome aforementioned obstacles, such as WZ4002, rociletinib (CO1686), osimertinib (AZD9291), and Almonertinib (HS-10296) (14–17). Based on the AURA trials, osimertinib is currently a standard of care for *EGFR*-mutant NSCLC patients with acquired resistance to first- or second- generation EGFR-TKIs owing to the T790M mutation (18–21). Moreover, the phase III FLAURA trial provides another option for first-line treatment of patients with *EGFR*-activating mutations, which showed the superiority of osimertinib over first-generation EGFR TKIs as a first-line treatment (22). However, despite their efficacy, acquired resistance will also eventually emerge. It seems that standard chemotherapy is the only way to go after osimertinib resistance, but novel strategies, such as newer generation TKIs and immune checkpoint inhibitors (ICIs), have been emerging, and a variety of combined strategies have been explored to optimize all treatment lines in recent years, as will be discussed later.

This review summarizes the current mechanisms of acquired resistance to osimertinib and discusses the promising strategies to manage such problems on the basis of rationalities and controversies in the transition from preclinical investigation to clinical practice. We highlight clues and challenges regarding future combination therapeutic options in treatment of *EGFR*-mutant NSCLC and put emphasis on ICIs, wishing to give some references to clinical practice.

## Evolution of TKIs to Third-Generation

### Osimertinib

Multiple third-generation EGFR TKIs were reported to inhibit T790M mutation, while exhibiting activity against *EGFR* ex19del and L858R mutation and sparing the inhibition of wild-type receptors (14–17). Compared to the other inhibitors, osimertinib has shown great superiority and was the only one approved by the Food and Drug Administration (FDA) and European Medicines Agency (EMA) to date (18–23). The initial phase I/II study AURA and extensive phase II study AURA2 demonstrated impressive and exciting responses of osimertinib in *EGFR*-mutant advanced NSCLC patients previously treated with EGFR TKIs (first- or second-generation), especially in patients with T790M mutation (19, 20). The subsequent phase III study AURA3 continued to confirm advantages of osimertinib both in efficacy and toxicity profile in *EGFR* T790M mutation-positive advanced NSCLC patients who progressed on first-line EGFR TKI therapy, in comparison to platinum plus pemetrexed chemotherapy (21). Then, the phase III trial FLAURA of osimertinib in previously untreated, *EGFR* mutation-positive (ex19del or L858R), advanced NSCLC compared to standard EGFR TKIs suggested its efficacy at delaying acquired resistance with a median progression free survival (PFS) of 18.9 months and overall survival (OS) of 38.6 months and less adverse events of grade 3 or higher in first-line therapy (22). Hence, gefitinib, erlotinib, afatinib, dacomitinib, and osimertinib have been recommended as first-line treatment by NCCN (category 1) (18).

In general, all EGFR TKIs play their respective values in clinical practice. For example, improvements were detected in brain metastases under the role of afatinib and osimertinib both in preclinical and clinical studies, while other EGFR TKIs produce limited efficacy (23, 24). Besides, osimertinib is the first choice for patients with primary T790M mutation (16, 22). In terms of uncommon mutations excluding the exon 20 insertion (ex20ins), osimertinib demonstrated favorable activity with a high response rate, an encouraging PFS, a long duration of overall response (DOR) and manageable toxicity (25). Nevertheless, only a small number of patients were assessed and objective response rates (ORRs) across specific uncommon mutation type were quite diverse with osimertinib treatment (25). Available data regarding the efficacy of first- or second-generation TKIs in NSCLC patients with uncommon *EGFR* mutations are inconsistent resulting from retrospective or *post hoc* analyses. In a *post hoc* analysis of afatinib data from the LUX-Lung 2, LUX-Lung 3, and LUX-Lung 6 trial populations reported by Yang et al., ORR was 71% and PFS was 11 months among patients with uncommon *EGFR* mutations with afatinib treatment, except for those with the T790M or ex20ins mutation (10). The third most common rare mutation is exon 20 S768I mutation, whose clinical data are inconsistent among studies. Cho et al. demonstrated an ORR of 38% with a PFS of 12.3 months in patients with just the S768I mutation (25). However, Chiu et al. reported an ORR of 100% and median PFS was 14.7 months with afatinib in patients with only the S768I mutation (26). In conclusion, both afatinib and osimertinib showed relatively high efficacy in uncommon *EGFR* mutations and can be considered as a treatment option based on current data. More studies with large number of patients are needed, and we must give a synthetic consideration in drug choice, such as activity, toxicity, mutation type and CNS metastasis. On the other hand, in view of ultimate drug resistance of osimertinib, more studies need to be conducted to compare benefits of latter-line to front-line. We also need to recognize that quite a few patients may not have the opportunity to take a biopsy again and change drugs after drug resistance.

### Other Prospective Third-Generation Drugs

Almonertinib has been approved recently by National Medical Products Administration (NMPA) in China, exhibited a median PFS of 12.3 months, acceptable toxicity and an ORR of 68.9% in second-line treatment of EGFR-mutant NSCLC after drug resistance in the phase II APOLLO trial (17). Lazertinib (YH25448), another mutant-selective third-generation EGFR inhibitor, was more effective and better tolerated than osimertinib in preclinical data (27). A phase I–II study (NCT03046992), enrolling patients with advanced NSCLC harboring activating mutations of *EGFR* who had progressed after EGFR TKI therapy, had further proved its tolerable safety profile and promising clinical activity (28). A phase 2 dose extension part is ongoing. Additionally, Cho et al. have recently reported the preliminary result of the phase I CHRYSALIS study (NCT02609776) that the ORR of 23 Part 1 patients receiving the combination of amivantamab (JNJ-61186372, EGFR-MET bispecific antibody) with lazertinib was 43.5%, and the safety profile was manageable (29). Abivertinib (AC0010) and alflutinib (AST2818) are also promising third-generation drugs, and we expect more results.

## Acquired Resistance to Third-Generation EGFR TKIs

**Figure 1:**
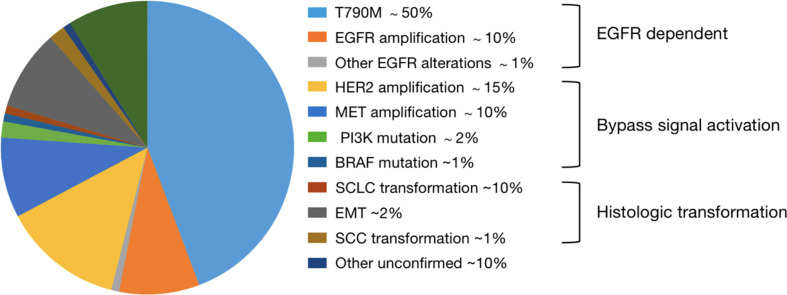
Mechanisms of acquired resistance to first- and second-generation EGFR TKIs.

Mechanisms of resistance to first- and second-generation EGFR TKIs have been well researched (**Figure 1**). Compared to previous generations, quite a few resistance mechanisms remain unknown and deserve further study. Besides, *EGFR* T790M mutation alone rarely induces resistance to osimertinib. Resistance occurs with a PFS of 18.9 months by treatment of osimertinib used in first-line, including EGFR-dependent and EGFR-independent resistance (22, 30–32). A study of mechanisms of acquired resistance to osimertinib given initially or at relapse in patients with *EGFR*-mutant NSCLC identified on next generation sequencing (NGS) from tumor tissue, presented at ASCO 2019, reported that resistance mechanisms to initial and later-line osimertinib are distinct from each other (33). Firstly, 59% of resistance mechanisms to first-line osimertinib were uncertain, but such part only made up 25% in patients receiving later-line osimertinib (33–35). Then, only 7% of patients receiving initial osimertinib developed EGFR-dependent resistance, contrary to a proportion of 34% in second-line (33–35). Additionally, histologic transformation/phenotypic change, including squamous cell carcinoma (SCC) histologic transformation, small-cell lung cancer (SCLC) histologic transformation and epithelial-to-mesenchymal transition (EMT), was a dominant resistance mechanism, particularly in first-line setting, and they suspected that EGFR-independent resistance may emerge earlier than EGFR-dependent resistance (33–36). Of course, some resistance mechanisms in early stages of research may play a part both in front-line and latter-line settings, and we have difficulty in classifying them precisely.

### First Line

Among the EGFR-dependent resistant population, C797S/G and L718Q take up a high proportion, followed by some other rare mutations, such as G724S, S768I, E709K, L692V and L798 (30–32, 34). The frequency of the C797S mutation was 7%, a tertiary mutation in exon 20 of *EGFR*, the second most frequent mechanism, only behind MET amplification, when osimertinib was given in first-line (34). Such mutation has been not only detected in osimertinib resistant patients, but also in patients with *EGFR* T790M treated with rociletinib, olmutinib, and narzatinib (30–35, 37). Given that the mutation is located in the kinase-binding site, it possibly abrogates the binding activity of osimertinib to EGFR (30–34).

Among the EGFR-independent mechanisms of resistance to third-generation TKIs, *MET* amplification, *HER2* amplification, amplifications of genes involving receptors [such as Insulin-like growth factor 1 receptor (IGF1R)], mutations or amplifications of genes involved in MAPK-PI3K signaling cascades (such as BRAF) and histologic transformation/phenotypic change have been reported (30–34). When osimertinib was administered as a front-line therapy, *MET* amplification ranked first, detected in 15% of patients by next-generation sequence ctDNA analysis (34). Moreover, *MET* amplification could also represent a potential mechanism of intrinsic resistance to osimertinib (38). *HER2* amplification occurred in 2% of cases and has been reported in one patient who experienced intrinsic resistance to osimertinib (34, 38). In terms of MAPK-PI3K pathway activation, *KRAS* G12D has been reported after disease progression both in first-line and subsequent lines of therapy (33–35). Then, the *BRAF* V600E mutation has been identified as a resistance mechanism to osimertinib in 3% of cases both in first- and latter-line therapy (33–35). *PIK3CA* mutations E453K, E545K, and H1047R were identified in six cases, with E545K being the most represented in 4% of cases, and *HER2* mutation was detected in 1% of cases, which is different from *HER2* amplification (34, 39).

**Figure 2:**
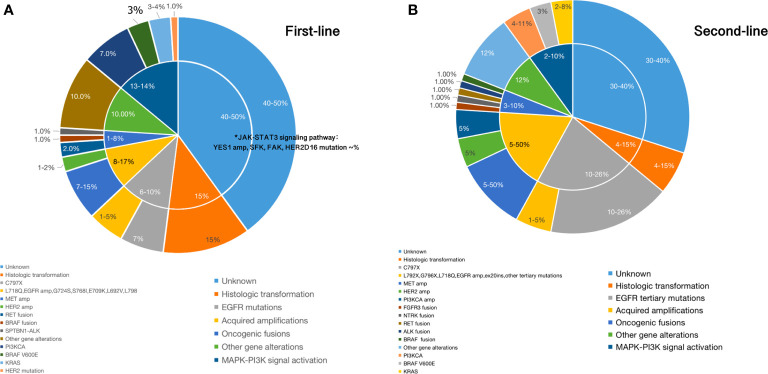
**(A)** Main mechanisms of resistance to first-line setting of third-generation EGFR TKIs; **(B)**. Main mechanisms of resistance to second-line setting of third-generation EGFR TKIs.

**Figure 3:**
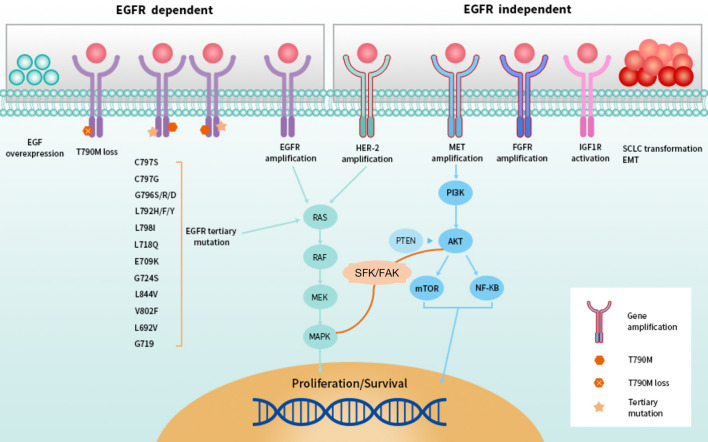
EGFR-dependent and -independent resistance to second-line setting of third-generation EGFR TKIs.

*HER2* alterations have been reported as resistance mechanisms to osimertinib, including amplifications and mutations, which are distinct molecular targets (39). However, they are mainly in-frame exon 20 insertions. In a case reported by Hsu et al., an exon 16 skipping *HER2* deletion (HER2D16), inducing resistance to osimertinib in a patient who is *EGFR* T790M-positive, was presented (40). The HER2D16 alteration has only previously been reported in breast adenocarcinoma, in which it was recognized to activate Src kinase signaling in approximately half of *HER2*-positive breast cancers (41). Different from breast cancers, expressing HER2D16 generates osimertinib resistance to *EGFR* T790M/L858R-mutant NSCLC cells in a *Src*-independent fashion through Src-bypass signaling, which was insensitive to Src inhibition with or without osimertinib (40, 41). They revealed that combined osimertinib and pan-HER small molecular inhibitor, afatinib, may synergistically overcome resistance to osimertinib in H1975-HER2D16 cells (40). Of note, Ichihara et al. reported that Src family kinases (SFK) and focal adhesion kinase (FAK) can sustain AKT and MAPK pathway signaling under continuous EGFR inhibition in osimertinib sensitive cells and inhibiting either the MAPK pathway or the AKT pathway enhanced the effects of osimertinib (42). Amplification of *YES1*, encoding SFK member YES1, has been reported as a resistance mechanism to osimertinib in NSCLC cell lines (42, 43). In addition, YES-associated protein (YAP) is the main mediator of the Hippo (also known as the Salvador–Warts–Hippo) signaling pathway, overexpression of which in NSCLC is associated with cancer progression, drug resistance, metastasis and poor prognosis (42–45). Another emerging key player involved in osimertinib intrinsic resistance is the RTK Anexelekto (AXL), which can interact with other RTKs, including EGFR and HER3, and sustain survival of tumor cells exposed to osimertinib (38, 46). Overexpression of *AXL* is also linked to EMT-associated resistance to osimertinib (44–46). Acquired oncogenic fusions and acquired cell cycle gene alterations occurred in 1–8 and 10% respectively (34). To date, nearly 50% of resistant mechanisms in first-line are still not clear. Main mechanisms of resistance to first-line use of third-generation EGFR TKIs were summarized in **Figure 2A**, and we and we present them more vividly in **Figure 3**.

### Second Line

Resistance will also inevitably happen after using third-generation EGFR TKIs in second-line, EGFR*-*dependent or -independent (**Figure 2B**). *EGFR* tertiary mutations, T790M loss, *EGFR* amplification, and EGF overexpression are EGFR-dependent alterations. *EGFR* C797S/G, G796S/R/D, L792F/H/Y, L798I, L718Q, E709K, G724S, L844V, V802F, L692V, G719 have been reported as *EGFR* tertiary mutations to cause acquired resistance to second-line use of osimertinib (30–32, 35, 47–50). The most common tertiary *EGFR* mutation is *EGFR* C797S, which occurs in exon 20 and accounts for 10–26% of cases of resistance to second-line osimertinib treatment (21, 35, 47, 48). Besides C797X mutations, a number of other rare point mutations in *EGFR* listed above have also been identified. Moreover, in second-line treatment, amplification of wild-type *EGFR* allele in addition to the presence of the *EGFR* ex19del allele was detected as a novel mechanism of resistance (49). Ex20ins mutation has also been reported in one patient after failure of second-line osimertinib therapy, and other mutations within exon 20 occurring after progression to osimertinib haven’t been established yet (35).

In terms of EGFR-independent resistance, alterations including *MET*, *HER2*, and fibroblast growth factor receptor (FGFR) amplification, bypass signal activation including RAS-MAPK/ERK and PI3K–AKT pathway and histologic transformation/phenotypic change have been verified (30–33, 35, 38, 49–51). In the AURA3 study, the loss of T790M was experienced by 43% of patients, whereas 57% retained the mutation (35). Osimertinib has demonstrated superior efficacy against *HER2* amplification through inhibiting downstream signaling pathway targets in genetically modified mice models in a preclinical trial (48, 51). However, activation of *HER2* or *MET* was also identified in osimertinib resistant patients (30–33, 35). Overexpression of HER2 or MET in cells can persistently activate ERK and AKT as a result of sharing part of the same downstream pathways as EGFR (30–33, 35, 50). *MET* amplification can occur with or without loss of the T790M mutation in second-line setting of osimertinib, which was observed in nearly 19% of the samples at disease progression (35). Also, *MET* amplification co-occurred with *EGFR* C797S in 7% of cases (35). Different from MET, HER2 can indirectly activate PI3K and amplification of *HER2* appears to mutually exclusive with T790M in *EGFR*-mutated NSCLC patients who develop resistance (35, 49, 50). *HER2* amplification has been identified in 5% of patients who have acquired resistance to second-line osimertinib (35). The combination of osimertinib and trastuzumab–emantisine has previously been shown to overcome *HER2* amplification-mediated resistance in *EGFR* T790M-positive NSCLC cell lines (52). Osimertinib combining with c-Met inhibitors, such as crizotinib, has been found effective in osimertinib-resistant *EGFR*-mutated NSCLC patients harboring *MET* amplification and more combination strategies will be discussed later (38, 53). Other resistance mechanisms to second-line osimertinib are similar to first-line, which may be presented in different proportions. We list them in **Figure 2B**.

## Treatment Options After Resistance of Third-Generation EFGR TKIs

**Figure 4:**
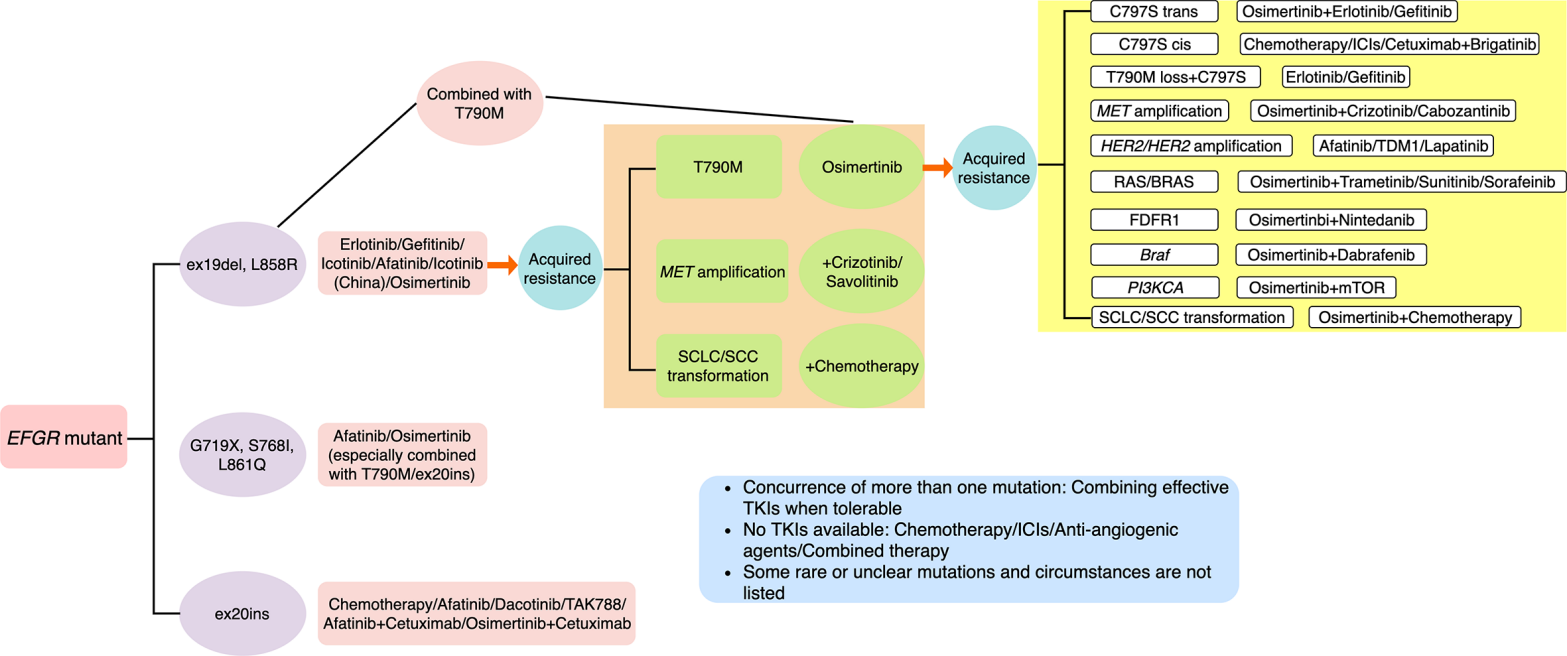
Summary of major resistance mechanisms to osimertinib and coping strategies which we found in literature. Some rare or unclear mutations and circumstances are not listed.

Just like resistance of first- and second-generation EGFR TKIs, progress after drug resistance can be divided into oligosis and broad progress (18, 31). Continuation of EGFR TKIs plus local treatment is the first recommendation if oligosis (18). Re-biopsy should be adopted to identify the exact mechanism of acquired resistance to direct latter treatment. However, successful tissue biopsy is often not feasible, which is an invasive procedure associated with morbidity and may be limited by patient refusal, insufficient sample for molecular testing, tumor location, and performance status of patients (54, 55). Moreover, misdiagnosis may be caused by intra-tumor heterogeneity and spatiotemporal variation within the same patient (56). Thus, T790M testing at clinical progression on a first-line EGFR TKI, using plasma circulating tumor DNA (ctDNA) testing has been recommended for its advantage of being non-invasive, which is also referred to as liquid biopsy (57–59). The viable approach has also enabled serial monitoring with repeated molecular analyses at multiple time points. In addition to ctDNA, cell-free DNA (cfDNA) and circulating tumor cell (CTC) in peripheral blood are also tumor-derived resources for liquid biopsy, which can be detected by various methods, including NGS, cobas EGFR mutation test, therascreen EGFR amplification refractory mutation system (ARMS), droplet digital polymerase chain reaction (ddPCR) and bead, emulsion, amplification and magnetics (BEAMing) (58–61). However, high rates of false negative ctDNA T790M have been observed across the AURA clinical trials with positive percentage agreement (PPA) of 51% in AURA3 (62). Based on this, it is recommended to repeat a tissue biopsy for patients with a negative plasma T790M result when feasible (57, 63). Of note, Sequist et al. reported that six patients who were directly treated with osimertinib after rociletinib resistance achieved partial response (PR) or stable disease (SD), and three patients with central nervous system (CNS) progression during rociletinib treatment received good control of CNS lesions after receiving osimertinib treatment (64). It suggests that rociletinib resistance may be due to incomplete targeted inhibition, and osimertinib can reverse this resistance, including CNS progress (64). In several clinical cases, platinum-based doublet chemotherapy was used to treat osimertinib-resistant patients with SCLC histologic transformation (30). Compared with osimertinib-sensitive cells, osimertinib-resistant cells with SCLC histologic transformation were more sensitive to paclitaxel (30). This finding suggested that paclitaxel might be a favorable option for osimertinib-resistant patients harboring SCLC histologic transformation (30). In addition to these, newer generation EGFR TKIs and combination therapies have become the most promising options. We have summarized major coping strategies which we found in literature in **Figure 4**.

## Newer Generation EGFR TKIs

To overcome acquired resistance of third-generation EGFR TKIs, an allosteric inhibitor EAI045 targeting T790M and C797S mutation was reported in Nature in 2016 (65). It binds to the allosteric sites in the tyrosine kinase molecule to change the conformation of the enzyme, thereby inhibiting the enzymatic reaction (65, 66). EAI045 has different potency for the two subunits of EGFR asymmetric dimer, and cannot inhibit dimerization-mediated signal activation (65, 66). EAI045 single drug has a weak inhibitory effect, but can make tumor remission in the rat lung cancer model when combined with cetuximab (65, 66). In addition, EAI045 combined with cetuximab is effective for L858R/T790M, but has no inhibitory effect on ex19del/T790M, which is mainly due to the fact that L858R can expand and the allosteric domain of tyrosine kinase, while 19del prevents the opening of the allosteric domain of the enzyme molecule (65, 66). The researchers call this phenomenon mutation specificity. These data were limited to laboratories, and whether they can be translated into clinical benefits still needs further research. Recently, other fourth generation EGFR TKIs EAI001 and JBJ-04-125-02 were reported, and more clinical trials are needed to evaluate their efficacy and safety profiles (66, 67). Furthermore, the aforementioned amivantamab (JNJ-61186372) is also a fourth generation EGFR TKI, which has shown preclinical activity in TKI-sensitive *EGFR*-mutated NSCLC models and in the ongoing CHRYSALIS study (68). Then, Cho et al. have also characterized the antitumor activity of amivantamab in multiple preclinical models harboring *EGFR* exon20ins mutations (29).

## Conventional Combinations of Third-Generation EGFR TKIs and Other Agents

Before ICIs were added, there were many traditional combination options with EGFR TKIs, including chemotherapy, other inhibitors, anti-EGFR monoclonal antibodies and anti-angiogenic target drugs. Although superior efficacy of combination of EGFR TKIs and chemotherapy was revealed, there existed controversies in the efficacy of EGFR TKIs plus chemotherapy in EGFR TKI-resistant NSCLC patients in clinical trials on previous generations of EGFR TKIs (69–71). In the mass, no apparent survival benefits were seen in such mode. Safety data were reported of an ongoing Japanese phase II trial of osimertinib with carboplatin/pemetrexed, and a phase I study of osimertinib with platinum and etoposide is currently recruiting (NCT03567642) (72). We are not optimistic about the results.

Whether the C797S and T790M mutations are in the same allele makes important biological significance. *In vitro* studies have reported C797S and T790M mutations in trans (on separate alleles) are sensitive to a combination of first- and third-generation TKIs whereas if C797S and T790M mutations are both in cis (on the same allele), EGFR TKIs alone or in combination are ineffective (33–35, 37, 47, 48). Based on this, combination of first-generation TKIs and osimertinib can overcome resistance of osimertinib when the C797S and T790M mutations are in different alleles (47, 48). Combination therapy trials of Nazartinib with gefitinib (NCT0329213, NCT03333343) and of osimertinib with gefitinib (NCT03122717) and dacomitinib (NCT03810807) are currently underway to verify the hypothesis that combining first- and third-generation EGFR TKIs may delay the C797S and T790M resistance mutations. Combinations of third-generation EGFR TKIs and other targeted agents were attempts based on heterogeneous resistance mechanisms. Cell experiments have shown that osimertinib combined with MEK inhibitor selumetinib can delay the emergence of drug resistance, and a phase I trial (NCT03392246) of osimertinib combined with selumetinib in the treatment of *EGFR-*mutant advanced NSCLC patients is ongoing. Furthermore, the TATTON study (NCT02143466) has reported recently that combination of osimertinib and savolitinib (AZD6094) showed acceptable risk-benefit profile and encouraging anti-tumor activity in patients with *EGFR* mutation-positive, *MET-*amplified, advanced NSCLC, who had disease progression on a previous EGFR TKIs (73). Based on data from TATTON, the SAVANNAH study (NCT03778229) further evaluating the combination of osimertinib and savolitinib in patients with *MET*-driven resistance to osimertinib is ongoing. In addition, the multi-drug, biomarker-directed, phase II platform ORCHARD trial (NCT03944772) is ongoing and evaluating resistance mechanisms and combination treatment options for patients with *EGFR-*mutant NSCLC who have progressed on first-line osimertinib therapy, including osimertinib and savolitinib, for patients with acquired *MET* amplification. The potential of osimertinib and savolitinib as a novel treatment option for patients with acquired *MET* amplification who are *EGFR* mutation-positive will be better assessed by these subsequent phase II studies. To date, numerous combined strategies with third-generation EGFR TKIs such as trastuzumab emtansine (T-DM1), JAK1 inhibitor, AXL inhibitor and BRAF V600E inhibitor are currently investigated in clinical trials (52, 74, 75). Inhibition of AXL kinase activity restored both *CDH1* expression and sensitivity to EGFR TKIs (44, 45). A recent research study revealed that AXL overexpression was associated with a poor response to osimertinib, whereas combination treatment with an AXL inhibitor and osimertinib prevented the development of intrinsic resistance to osimertinib and the subsequent emergence of resistant clones *in vitro* and *in vivo (* 46). Therefore, AXL remains a promising next line therapeutic target for EMT-associated resistance, with several pharmacological inhibitors in early clinical development (ClinicalTrials.gov identifiers NCT03255083, NCT02424617, NCT02729298). As far as we are concerned, strategies to overcome resistance to osimertinib should be individualized for diverse mechanisms on the basis of tissue or liquid biopsy. Meanwhile, we have to say that such combinations may make limited overall benefits in the general population before the arrival of an efficient newer generation of EGFR TKIs and how to add ICIs may be a better direction of efforts.

## ICIs

### Argument About the Interactions of the Oncogenes and Tumor Microenvironment

ICIs, hot representative of immunotherapy, are epoch-making in treatment of NSCLC, benefiting from the study of the tumor microenvironment (TME). However, at present, ICIs are not suitable for everyone, and patient selection for ICIs depends mainly on some molecular biomarkers currently, such as programmed cell death protein 1 (PD-1), programmed death-ligand 1 (PD-L1), tumor mutational burden (TMB), CD8+ T cell infiltration, Tim-3, and so on (76–78). Even so, known markers are not reliable enough to predict the efficacy yet. In recent years, several ICIs have been approved in patients with negative driver genes, PD-L1 expression or without screening, but the performance in the positive driver genes seems to be unsatisfactory (79). PD-L1 expression has been reported to be associated with *EGFR* mutations in NSCLC (79–84). In murine NSCLC models, EGFR signaling induced by *EGFR* mutations activated PD-L1 expression and induced immune escape, and PD-L1 expression was down-regulated by EGFR TKIs treatment (81). In theory, the rapid release of antigen through dying tumor cells under TKIs could enhance the inflammatory response (82). The data published by Wu showed that the proportion of primary resistance to EGFR TKIs was higher in PD-L1-positive patients, suggesting that these patients may benefit from ICIs (83). In other words, strong PD-L1 expression predicted a poor response to EGFR TKIs and might be associated with *de novo* resistance to targeted therapy in first line therapy (84). However, others hold conflicting views (85). Similarly, conflicting results have been obtained in clinical trials of combining ICIs with EGFR TKIs in treatment of NSCLC. It’s worth mentioning that it’s also possible that there may be no relationship between the oncogenes and TME, or multiple oncogene mutations are needed to predict efficacy of ICIs. Concept of genomic mutation signature (GMS) consisting of eight genes was proposed as a better predictive tool lately, which needs further research (86). In addition, serious side effects are often seen in *EGFR-*mutant NSCLC patients treated with ICIs, which might arise from interactions of the oncogenes and TME. Clarifying the relationships between them and lessening adverse reactions deserve further investigations. We have summarized studies on efficacy and safety profiles of ICIs in *EGFR*-mutant NSCLC patients in **Table 1**.

**Table 1:** Efficacy and safety profiles of immune checkpoint inhibitors in *EGFR*-mutant non-small cell lung cancer patients.

Category	Study		Therapy			Efficacy	Safety	Reference
Immune monotherapy	CheckMate 012 (First-line)		O	I	8 (15%)	ORR 14 *vs* 30% (Wildtype). 24 weeks PFS 14% vs. 51% (Wildtype)	Tolerable	(87)
	KEYNOTE-001 (Previously treated)		K	I	74 (17%)	mOS 6.0 *vs*. 11.9 months 5-year OS 7.9 *vs.* 16.4%	Tolerable	(88)
	NCT03513666		K	II	–	No response	–	(89)
	BIRCH	No CT	T	II	13 (11%)	ORR 23 *vs* 19% (Wildtype*).*	Tolerable	(90)
		1 CT			18 (8%)	ORR 0 *vs*. 21%		
		≥2 CT			14 (7%)	ORR 7 *vs*. 35%		
	CheckMate 057 (Second-line)		O	III	82 (14%)	HR 1.18 (0.45–2.07) *vs*. 0.66 (0.51–0.85) (Docetaxel)	–	(91, 92)
	KEYNOTE-010 (Second-line)		K	II/III	86 (8%)	HR 0.88 (0.45–1.72) *vs*. 0.66 (0.55–0.79) (Docetaxel)		
	POPLAR (Second-line)		T	II	18 (6%)	HR 0.99 (0.29–3.40) *vs*. 0.70 (0.47–1.04) (Docetaxel)		
	OAK (Second-line)		T	III	85 (10%)	HR 1.24 (0.71–2.18) *vs*. 0.69 (0.57–0.83) (Docetaxel)		
Third-line or later	ATLANTIC		I	II	111 (*EGFR*+/*ALK*+)	(PD-L1 ≥25%) ORR 12.2% (95% CI 5.7 to 21.8) mPFS 1.9 months mOS 13.3 months	Tolerable	(93)
Immunization combined with EGFR TKIs	TATTON		I+OSI	I	–	–	Severe	(94)
	CAUREL		I+OSI	III	–	–	Severe	(95)
	CheckMate 012		O+Erlo	I	20 (TKI treated)	ORR 15% (1 CR), DCR 65%, No PD-L1 ORR 0		(96, 97)
					1 (TKI untreated)	2.2 months PR CR 27 months		
Immunization combined with chemotherapy and anti-angiogenic therapy	A retrospective study		O+CT	–	5	45.5% mPFS 7.47 months 1 year OS 54.5%	Tolerable	(98)
			K+CT	–	6			
	IMpower150		ABCP	III	35 (8.8%)	mOS 29.4 months	Tolerable	(99, 100)
			BCP		45 (11.3%)	mOS 18.1 months		
	–		Toripalimab+CT	II	–	50% mPFS 7 months	Tolerable	(101)
	KEYNOTE-789		K+CT	III	–	Ongoing	–	(102)
	CheckMate 722		O+CT	III	–	Ongoing	–	(103)
	WJOG8515L		O	II	–	Ongoing	–	(104)
Combined immunity	CheckMate 012		O+Ipili	I	8 (10.4%)	ORR 50%	Tolerable	(105)
	KEYNOTE-021		K+Ilili	I	10 (22%)	ORR 10%	Tolerable	(106)

O, Nivolumab; K, Pembrolizumab; T, Atezolizumab; I, Durvalumab; Ipili, Ipilimumab; Erlo, Erlotinib; OSI, Osimertinib; CT, Chemotherapy; ABCP, Atezolizumab plus bevacizumab plus carboplatin plus paclitaxel; BCP, Bevacizumab plus carboplatin plus paclitaxel; ORR, Objective response rate; OS, Overall survival; PFS, Progression free survival; mOS, Median OS; mPFS, median PFS; CR, Complete response; HR, Hazard ratio.

### ICI Monotherapy

Data derived from subgroup analysis of large clinical trials have revealed that different agents seemed to have different impacts, but in general, the clinical activity of ICI monotherapy in first-line treatment of *EGFR-*mutant NSCLC was limited (87–90, 107). More studies focused on the role of ICIs in second-line treatment of *EGFR-*addicted NSCLC patients after TKIs resistance, which we also concern about. The IIIb/IV safety trial CheckMate 153 of nivolumab in patients with advanced or metastatic squamous or non-squamous NSCLC who received at least one prior line found that partial response rate was only 11% (n = 55) in the *EGFR-*mutated subgroup compared with 16% (n = 300) in the *EGFR* wild-type subgroup (108). A meta-analysis assessing the role of ICIs (nivolumab, pembrolizumab and atezolizumab) as second-line therapy in *EGFR-*driven advanced NSCLC, including three trials (CheckMate 057, KEYNOTE-010 and POPLAR study), concluded that *EGFR-*mutant patients didn’t benefit from ICIs over docetaxel in terms of OS (91). Another systematic review and meta-analysis of four trails (OAK was added) drew the same conclusion (92). Indeed, these studies have their limitations as *EGFR* mutation was not determined by centralized testing, but reflect some of the truth. Of note, the phase II trial ATLANTIC evaluated the effectiveness of durvalumab in second-line and above treatment of patients with locally advanced or metastatic *EGFR*-mutant NSCLC who had experienced at least two regimens of chemotherapy or EGFR TKIs (93). In patients with *EGFR+/ALK+* and ≥25% tumor cells expression of PD-L1, the ORR and median OS were 12.2% and 13.3 months, which were significantly better lpagthan chemotherapy (93). As a whole, although durvalumab has revealed a distinct advantage compared to chemotherapy in ATLANTIC trial, *ICIs* haven’t achieved a desired effect in treatment of *EGFR-*mutant NSCLC, neither first- nor second-setting.

### ICIs Combined With EGFR TKIs

Based on the unsatisfactory efficacy of monotherapy in patients with *EGFR* mutations, whether *ICIs* can become a successful assistant of TKIs in combination mode has become a hot topic. D’Incecco et al. reported that elevated PD-L1 levels are associated with *EGFR* mutations and EGFR TKIs treatment, suggesting that the combination of anti-PD-1/PD-L1, and EGFR TKIs might have synergistic effects in NSCLC therapy (80). The TATTON study showed that treatment with durvalumab combined with osimertinib in treated patients with *EGFR* mutations was associated with an increased risk of poisoning (94). The phase III clinical trial CAUREL comparing the effects of durvalumab combined with osimertinib with osimertinib alone in T790M-positive NSCLC patients who have been treated with EGFR TKIs and found that the incidence of EGFR TKI-associated interstitial pneumonitis in both EGFR TKI and nivolumab cohort was much higher than TKI single drug cohort (95). More, a longer PFS (2.1 months) in the T790M-negative patients than the T790M-positive patients (1.3 months) was seen, and the former also had a higher proportion of tumors with a PD-L1 expression along with higher CD8+ tumor infiltration and TMB (95). Many other combination strategies with addition of ICIs to targeted TKIs were proven to induce significant treatment-related toxicities without significantly improved efficacy (109–111). As part of phase I clinical trial CheckMate 012, twenty who have received with erlotinib and one TKI-naive *EGFR-*mutant NSCLC patients were treated with nivolumab plus erlotinib, and the trial concluded that nivolumab plus erlotinib was tolerable and made durable responses in *EGFR-*mutant, TKI-treated NSCLC patients (96, 97). In conclusion, combined treatment of EGFR TKIs and ICIs is still at an early stage, and further efforts to assess different combinations are necessary. At present, given that the efficacy of combining PD-1/PD-L1 inhibitors with EGFR TKIs as an option for *EGFR*-mutant advanced NSCLC patients is still not very clear, and serious adverse reactions seem inevitable, clinicians need to weight the advantages and corresponding disadvantages when making decisions.

### ICIs Combined With Chemotherapy and Anti-Angiogenic Therapy

Broadly speaking, the efficacy and safety of ICIs combined with TKIs are not very satisfactory. Nevertheless, ICIs combined with chemotherapy and anti-angiogenic therapy have been a major research direction. ICIs combined with platinum-based chemotherapy have been the currently recommended first-line treatment for advanced *EGFR*-/*ALK-* NSCLC (18). A small retrospective study showed that ORR of combined treatment of ICIs and chemotherapy was 45.5%, median PFS was 7.47 months, and 1-year survival rate was 54.5% in patients treated by chemotherapy combined with pembrolizumab (6) and nivolizumab (5) on osimertinib resistance (98). Of course, the result still needs to be validated by large prospective trials for its small sample. The blockbuster study IMpower150 (NCT02366143) showed that the combination of atezolizumab and bevacizumab conferred synergistic efficacy to patients with metastatic non-squamous NSCLC in terms of PFS and median OS, regardless of *EGFR* or *ALK* aberration or the expression of PD-L1 (99). Socinski et al. have reported the final OS analyses from Impower150 for ACP (Arm A, atezolizumab + carboplatin + paclitaxel) *vs* BCP (Arm C, carboplatin + paclitaxel + bevacizumab) and found that in patients with *EGFR+/ALK+* tumors, OS was comparable in Arms A (ACP, atezolizumab + carboplatin + paclitaxel) and C (BCP, carboplatin + paclitaxel + bevacizumab) (97). However, continued OS benefit was seen in Arm B (ABCP, atezolizumab + carboplatin + paclitaxel + bevacizumab) *vs* C in these subgroups, and the safety profile of each regimen was consistent with previously reported data at the second interim OS analysis (99, 100). Noteworthy, Impower150 study is currently the only randomized, prospective phase III clinical trial to demonstrate efficacy of ICIs in oncogene-addicted NSCLC patients. Furthermore, a phase II study of toripalimab combined with chemotherapy has demonstrated a higher ORR of 50% and longer median PFS of 7 months in *EGFR*-mutant advanced NSCLC patients who failed to prior EGFR TKIs therapies, which provides a novel choice for such patients (101). The KEYNOTE-789, CheckMate 722, and WJOG8515L trials are ongoing enrolling advanced non-squamous NSCLC and *EGFR*-mutant patients who progressed on prior TKIs therapies, and patients were assigned to receive chemotherapy alone or combined with ICIs. We look forward to good results in the future (102–104).

### Combined ICIs

The current research data on the combination of ICI and ICI for *EGFR* mutation-positive patients are insufficient. CheckMate 227 has confirmed the long and lasting benefits in OS by combing nivolumab and ipilimumab (112). More, an open-label, multi-center, randomized phase III clinical study CheckMate-9LA has found obvious benefits in OS without new safety concerns in treatment of nivolumab plus ipilimumab plus chemotherapy (113). However, both of them excluded the presence of *EGFR* mutations or known *ALK* translocations. In CheckMate 012, eight patients with *EGFR* mutations without chemotherapy received nivolumab combined with ipilimumab, and the ORR was 50% (105). Cohort D and H of KEYNOTE-021 reported an ORR of only 10% in TKIs-pretreated *EGFR*-mutant NSCLC patients by combined treatment of pembrolizumab and ipilimumab (106). Thus, conclusions were inconsistent in terms of remission rate, and we must be careful of serious adverse reactions. More combinations need to be explored, especially for patients pretreated with osimertinib. It seems that how to reduce side effects in setting of ICIs is particularly important, either single or combined.

## Conclusions and Perspectives

Third-generation EGFR TKI osimertinib has confirmed its position both in first-line and second-line after resistance to previous generations of TKI treatment in *EGFR*-mutant advanced NSCLC patients. However, almost all patients will face resistance to osimertinib, and the mechanisms are complex and diverse, none of which exceeds 20%. In our view, although there are some other treatment options reported, three main strategies to cope with resistance to osimertinib are next generation EGFR TKIs, conventional combinations of EGFR TKIs and other agents and treatment combined with ICIs. Firstly, next generation EGFR TKIs EAI045, EAI001, JBJ-04-125-02 and amivantamab are in the research phase and need to be verified in more trials, but we have seen great potential. Then, benefiting from extensive study of mechanisms involved in resistance development to third-generation EGFR TKIs, we could combine osimertinib with chemotherapy, other oncogene inhibitors, anti-EGFR monoclonal antibodies and anti-angiogenic agents based on specific resistance mechanism. We imagine that future research will keep laying emphasis on resistance mechanisms to EGFR TKIs, and unknown mechanisms of resistance deserve urgent elucidation with third-generation agents widely used in the first line, primary or acquired. The last, to offer patients with oncogene addiction the chance of ICIs induced long-term control of disease, many novel combinations with ICIs have been explored and have made some breakthroughs. From our point of view, ICIs combined with chemotherapy and anti-angiogenic agents are by far the most promising mode, which may be due to release of antigen through killed tumor cells and deserve further study. Of course, given that serious adverse reactions have become a major obstacle in further promotion of many combination modes, we should strive to identify causes of such side effects and learn to lessen them. In general, further understanding of interactions of oncogenes and TME, relationships between multiple markers, a more accurate predictive model, novel combinations and control of adverse reactions are future directions of efforts. Of course, immunotherapy is not limited to ICIs, which has enormous potential to be tapped. Notably, biopsy again after resistance of third-generation EGFR TKIs is particularly crucial as its guiding role in next treatment. Based on this, it’s important to develop non-invasive biomarker tests persistently, which may impinge on a greater fraction of NSCLC patients.

## Author Contributions

LW determined the writing direction and was responsible for the manuscript writing and modification. LK was responsible for literature collection and collation. ZZ took part in making charts. JY gave ideas and suggestions on selecting directions. XM provided financial support and review and revise manuscript. All authors contributed to the article and approved the submitted version.

## Funding

This work was supported by National Natural Science Foundation of China (81972864).

## Conflict of Interest

The authors declare that the research was conducted in the absence of any commercial or financial relationships that could be construed as a potential conflict of interest.
